# Development of a contacting transwell co-culture system for the *in vitro* propagation of primary central nervous system lymphoma

**DOI:** 10.3389/fcell.2023.1275519

**Published:** 2023-11-27

**Authors:** Mayuko Nishi, Kensuke Tateishi, Jeremiah Stanleyraj Sundararaj, Yoko Ino, Yusuke Nakai, Yasuyoshi Hatayama, Yutaro Yamaoka, Yusaku Mihana, Kei Miyakawa, Hirokazu Kimura, Yayoi Kimura, Tetsuya Yamamoto, Akihide Ryo

**Affiliations:** ^1^ Department of Virology III, National Institute of Infectious Diseases, Tokyo, Japan; ^2^ Department of Microbiology, Graduate School of Medicine, Yokohama City University, Yokohama, Japan; ^3^ Department of Neurosurgery, Graduate School of Medicine, Yokohama City University, Yokohama, Japan; ^4^ Laboratory of Biopharmaceutical and Regenerative Science, Graduate School of Medical Science, Yokohama City University, Yokohama, Japan; ^5^ Advanced Medical Research Center, Yokohama City University, Yokohama, Japan; ^6^ Life Science Laboratory, Technology and Development Division, Kanto Chemical Co., Inc., Tokyo, Japan; ^7^ Research Center for Influenza and Respiratory Viruses, National Institute of Infectious Diseases, Tokyo, Japan; ^8^ Department of Health Science, Gunma Paz University Graduate School of Health Sciences, Takasaki-shi, Japan

**Keywords:** PCNSL, lymphoma, vitrigel, pericytes, HGF, Pin1, NFkB, NFAT

## Abstract

Primary central nervous system lymphoma (PCNSL) is a malignant neoplasm of the central nervous system that is refractory to treatment and has extremely poor prognosis. One factor hindering the development of therapeutic options for PCNSL is its molecular heterogeneity and the extreme difficulty in establishing *in vitro* cell lines that permit intensive research on this disease. In the present study, we developed a method to propagate PCNSL cells *in vitro* using a contacting transwell cell culture system involving brain vascular pericytes. The co-culture system was found to recapitulate the tumor microenvironment that is influenced by the biological activity of adjacent pericytes, and to sustain the survival and proliferation of PCNSL cells *in vitro*. We further delineated the underlying molecular mechanisms and found that the HGF–c-Met axis may be involved in the long-term *in vitro* culture of PCNSL cells. Moreover, the peptidylprolyl isomerase Pin1 was found to play a key role in PCNSL cell survival and it sustained proliferation through interactions with key transcription factors related to B-cell lymphomagenesis. These results suggest that our *in vitro* co-culture system is well suited to analyzing the biological and molecular characteristics of PCNSL, and may contribute to the discovery of new therapeutic interventions.

## Introduction

Primary central nervous system lymphoma (PCNSL) is a highly malignant lymphoma that is confined to the central nervous system and does not involve other organs. PCNSL accounts for 2%–4% of intracranial tumors, and its incidence has been rising in recent years. The age of onset is 45–80 years (median 60 years), with a predilection for the elderly (Villano et al., 2011). The prognosis is generally poor, with a 5-year survival rate of 30%–40% (Chihara et al., 2018). This is largely due to relapse, which has been observed in 35%–60% of patients 2 years after the initial diagnosis (Grommes et al., 2019). PCNSL generally presents with a histopathology of diffuse large B-cell lymphoma (Chapuy et al., 2018; Schmitz et al., 2018), regardless of Epstein–Barr virus (EBV) infection. Similar to lymphomas involving the lymph nodes, PCNSL is a molecularly heterogeneous disease with a variable response to standard therapy (Hernández-Verdin et al., 2023). In contrast with nodal lymphomas, the cerebral location and growth of PCNSL appear to be related to the tumor microenvironment that is characteristic of brain malignancies. Also, PCNSL has a worse prognosis because of its anatomical location. As the efficacy of drugs used to treat PCNSL depends on their bioavailability in the brain parenchyma, findings pertaining to the response of nodal lymphoma to innovative agents cannot be directly applied to the clinical treatment of PCNSL patients (Schaff and Grommes, 2022).

The development of cell culture platforms is essential not only for preclinical drug discovery, but also for analyzing the molecular mechanisms in cancer biology. To date, the screening of drugs for PCNSL and the elucidation of their molecular mechanisms have not been possible because of the difficulty in extracting PCNSL cells from patients and culturing them *in vitro*. Neither patient-derived xenograft (PDX) models nor primary PCNSL cell lines with adequate clinical information have been commercially available for PCNSL. We recently established original PDX models from consecutive patients diagnosed with PCNSL at Yokohama City University Hospital (Tateishi et al., 2020). Biopsy tumor specimens were stereotactically transplanted into the brains of young immunodeficient mice (SCID Beige) after cell processing, and brain tumorigenesis was evaluated. These models are useful for exploring tumor-related signaling factors as therapeutic target molecules, and for examining the possibility of precision medicine for PCNSL (Nakamura et al., 2016). However, it has been extremely difficult to propagate these PDX models *in vitro* because of the challenge in creating an *in vivo* tumor microenvironment.

Protein phosphorylation is a major post-translational modification that regulates a plethora of cellular processes, including cell proliferation, differentiation, and cell death (Lu et al., 2002). Peptidylprolyl *cis-trans* isomerase NIMA-interacting 1 (Pin1) is a regulator that specifically interacts with phosphorylated Ser/Thr-Pro (pS/T-P) motifs and catalyzes *cis*/*trans* isomerization, leading to conformational changes of its substrates that are involved in cancer development (Lu et al., 1996; Yaffe et al., 1997; Lu et al., 2002; Lu and Zhou, 2007). A recent study demonstrated that Pin1 is overexpressed in most cancers and contributes to tumor progression by mediating multiple signaling factors (Chen et al., 2018). Indeed, Pin1 was highly expressed in xenograft-forming PCNSL tumors, which enhanced tumor progression partially via NF-κB activation (Tateishi et al., 2020).

In this study, we developed a method for culturing patient-derived PCNSL cells *in vitro* using a contacting transwell cell culture system with brain vascular pericytes (BVPs). This method reconstructs the cell proximity in the microenvironment of the brain, allowing PCNSL cells to proliferate without phenotypic changes. By utilizing multi-omics analyses, we further attempted to identify intracellular signaling pathways and molecules associated with PCNSL survival and proliferation *in vitro*.

## Materials and methods

### Cell culture

HKBML, a human PCNSL-derived cell line, was provided by RIKEN Cell Bank (RIKEN BRC, RIKEN BioResource Center, Tsukuba, Japan) and cultured in RPMI 1640 (FUJIFILM Wako Pure Chemical Corporation, Osaka, Japan) supplemented with 10% fetal bovine serum (FBS) and 1% penicillin/streptomycin. Human BVPs were purchased from Angio-Proteomie (Boston, USA) and cultured in Pericyte Media (PromoCell, Heidelberg, Germany). Human astrocytes were obtained from Gibco (Waltham, MA, United States) and were cultured and maintained in astrocyte medium (Gibco). Human HUV-EC-C cells were purchased from JCRB Cell Bank (Osaka, Japan) and were cultured in Ham’s F12K medium with 10% FBS, 100 μg/mL heparin, and 50 μg/mL endothelial cell growth supplement. MRC5 cells were purchased from RIKEN Cell Bank (RIKEN BRC) and cultured in Dulbecco’s modified Eagle’s medium (DMEM) supplemented with 10% FBS and 1% penicillin/streptomycin. PCNSL cells were obtained from orthotopic xenograft models. Briefly, surgically removed patient tumor tissues were enzymatically dissociated and 1×10^5^ cells were stereotactically implanted into the right striatum of 4- to 9-week-old female SCID Beige mice (Charles River, Yokohama, Japan) within 6 h. Brain tumors were harvested when neurological symptoms became critical or after body weight loss of more than 20%. After tumor dissociation, cells were seeded on ad-MED Vitrigel 2 (KANTO CHEMICAL, Tokyo, Japan), on which BVPs were seeded in advance in RPMI 1640 supplemented with 2% FBS, 1× B27 supplement (Gibco), and 1% penicillin/streptomycin.

### *In vitro* ad-MED vitrigel cell culture

Option rings were placed on the reverse surface of ad-MED Vitrigel 2 and chambers were immersed in culture medium for 10 min at room temperature (Uzu and Takezawa, 2020). After the medium was removed, BVPs were seeded for 24 h on the reverse surface of the chambers at a cell density of 2×10^4^ cells per 24-well plate or 1×10^5^ cells per 12-well plate. After removal of the culture medium and option rings, the chambers were placed upside down in 24- or 12-well plates filled with 0.5 mL of PCNSL cell culture medium. PCNSL cells were suspended in 0.2 mL of the culture medium and seeded at a cell density of 5×10^3^ to 1×10^4^ cells per 24-well plate, and then cultured for 3–9 days.

### Cell viability analysis

PCNSL cells were co-cultured with or without BVPs for 4–6 days. Cells in vitrigel were pipetted with 200 μL of cell culture medium several times and collected in microtubes. Equal volumes of CellTiter-Glo Reagent (Promega Corporation, Madison, WI, United States) were added to the microtubes and mixed by vortex. After incubation at room temperature for 10 min, the cell lysate was transferred to the multi-well plate and luminescence was measured with a GloMax Microplate Reader (Promega Corporation, Madison, WI, USA).

### Immunocytochemistry

Mouse brains were fixed with 10% formalin, and paraffin-embedded tissue sections were examined by immunohistochemical analysis. The sections were subjected to antigen retrieval by autoclave in sodium citrate buffer (pH 6.0) at 108°C for 10 min, and then treated with 3% H_2_O_2_ in methanol. After blocking with Blocking One Histo (NACALAI TESQUE, Kyoto, Japan), sections were incubated with primary antibody at 4°C overnight. Color development was performed using the DAKO EnVision™ + Dual Link System-HRP system (Dako, CA, USA) and counterstained with Mayer’s Hematoxylin Solution (MUTO PURE CHEMICALS, Tokyo, Japan). The primary antibodies used in this study were as follows: anti-PDGFRβ (Proteintech, Chicago, IL, USA); anti-NG2 (Abcam, Cambridge, MA, United States); anti-CD20 and anti-CD10 (Leica, Wetzlar, Germany); anti-CD79a and anti-BCL6 (NICHIREI BIOSCIENCES, Tokyo, Japan); and anti-MUM1 (Dako).

### Glutathione S-transferase (GST) pull-down and immunoblot analysis

GST pull-down assays were performed as previously described (Nishi et al., 2011). Briefly, HKBML cells were lysed with GST pull-down buffer (50 mM HEPES pH 7.4, 200 mM NaCl, 10% glycerol, 1% Triton X-100, 1.5 mM MgCl_2_, 1 mM EGTA, 1 mM EDTA, 100 mM NaF, 1 mM Na_3_VO_4_, 0.5 μg/mL leupeptin, 1 μg/mL pepstatin, and 0.2 mM PMSF) and clarified by centrifugation. Cell lysates were then incubated at 4°C for 3 h with glutathione-Sepharose beads containing 5 μg of either GST or GST-Pin1. The collected beads were washed three times with GST pull-down buffer. The lysate proteins were mixed with SDS sample buffer and processed for SDS-PAGE. The proteins were transferred onto PVDF membranes (Millipore, MA, USA) and immunoblotting was performed as previously described (Miyakawa et al., 2018) using primary antibodies for NFATc2 (Santa Cruz Biotechnology, TX, USA), p65 (Cell Signaling Technology, MA, USA), c-Jun (Cell Signaling Technology), BCL6 (Proteintech), and HRP-conjugated secondary antibody (Roche Diagnostics, Mannheim, Germany).

### Phosphoprotein identification by liquid chromatography/tandem mass spectrometry (LC-MS/MS)

Phosphopeptides were enriched using Phos-tag Agarose beads (FUJIFILM Wako Pure Chemical Corporation) as described in a previous study (Ino et al., 2022a). Phosphoproteins were analyzed using an Orbitrap Q Exactive HF mass spectrometer (Thermo Fisher Scientific, Bremen, Germany) coupled with an UltiMate 3000 HPLC system (Thermo Fisher Scientific). To identify phosphoproteins, peak lists were created using Proteome Discoverer software (version 2.2, Thermo Fisher Scientific) and searched against human protein sequences in the UniProt Knowledgebase (UniProtKB/SwissProt, 2020) using the Mascot search engine (version 2.7.0; Matrix Science, London, United Kingdom). Search parameters were as follows: enzyme, trypsin, variable modifications, acetyl (protein N-term), oxidation (Methionine), carbamidomethyl (Cysteine), carbamyl (N-term), and phosphorylation (Serine, Threonine, and Tyrosine). A false discovery rate of less than 1% and a peptide score ≥30 were adopted as the acceptance criteria for identifications. Quantitative analysis of MS data was performed using Progenesis QI for proteomics software (version 2.0; Nonlinear Dynamics, Newcastle, United Kingdom). Ingenuity Pathway Analysis (IPA) software (content version: 62089861, Release Date: 2021-02-17; QIAGEN, Venlo, Netherlands) was used for the biological analysis.

### Ligand–receptor expression analysis

Ligand–receptor expression analysis was performed using a PrimerArray of human cytokine–cytokine receptor interaction (PH001, Takara Bio, Kusatsu, Japan) in combination with a Real-Time System CFX96 (Bio-Rad, Hercules, CA, USA). This PrimerArray is a set of real-time reverse transcription–polymerase chain reaction (RT-PCR) primers used for the analysis of mRNA expression. The array contains a mixture of 96 primer pairs for 88 target genes and eight housekeeping genes. Total RNAs of HKBML cells and BVPs were extracted using an RNA Prep Kit (KANTO CHEMICAL), and cDNAs were synthesized from 250 ng of total RNA. Real-time PCR reaction was performed according to the manufacturer’s instructions. Relative cDNA levels for the target genes were analyzed by the 2^−ΔΔCT^ method using GAPDH as the internal control for normalization (Jin et al., 2019). In choosing ligand–receptor combinations, mRNAs for soluble factors expressed in BVP were selected using expression level 1 as the threshold criterion, and then expressions of their corresponding receptors in PCNSL were investigated. Phosphoproteomic analysis was used as a reference.

### Quantitative PCR

HKBML cells were co-cultured with or without BVPs for 4–6 days. Cells in vitrigel were pipetted several times and collected in microtubes. After vitrigels were washed with PBS three times, RNA Prep reagent was added to the microtubes and to the reverse side of each vitrigel where BVPs were attached. HKBML cells and vitrigel were incubated at room temperature for 5 min, then mRNAs of HKBML cells and BVPs were extracted from the cell lysates using the RNA Prep Kit (KANTO CHEMICAL), and cDNAs were synthesized from 1 μg of total RNA samples. A 25-μL reaction was set up containing 1 μL of cDNA (equivalent to 50 ng), 2 μL of a mixture of forward/reverse primers (10 μM), 8.5 μL of RNase-free water, and 12.5 μL of TB Green *Premix Ex Taq* II (Takara Bio). Thermal cycling conditions were as follows: 95°C for 30 s for initial denaturation; 39 cycles at 95°C for 5 s and 60°C for 30 s; and a final 10-min denaturation step at 95°C. Relative cDNA levels for the target genes were analyzed by the 2^−ΔΔCT^ method using GAPDH as the internal control for normalization (Jin et al., 2019). The primer sequences used for HGF and MET, as shown in Supplementary Figure S5, were as follows: HGF-fwd 5′- AAA​GGA​CTT​CCA​TTC​ACT​TGC-3′, HGF-rev 5′- CGC​TCT​CCC​TTA​CTC​AAG​CTA-3′, MET-fwd 5′-GTG​CCA​AGC​TAC​CAG​T-3′, MET-rev 5′-CTT​CGT​ACA​AGG​CGT​CT-3’. Pin1-fwd 5′-ATC​AAG​TCG​GGA​GAG​GAG-3′, Pin1-rev 5′-GCG​TCT​TCA​AAT​GGC​TTC​T-3’.

### Lentiviral shRNA knockdown of Pin1

Plasmids encoding shRNAs targeting *PIN1* (TRCN0000001035 and TRCN0000010577) were obtained from Sigma-Aldrich (St Louis, MO, USA) and co-transfected with the packaging plasmid mix into 293 T-lenti-X cells using Lipofectamine 3000 (Thermo Fisher Scientific). The supernatants were filtered using 0.45-μm filters and mixed with the Lenti-X Concentrator (Clontech Laboratories, CA, United States), and incubated for 3 h at 4°C. The mixture was centrifuged, and the pellet was resuspended with PBS and then added to HKBML cells overnight, followed by selection using 2 μg/mL puromycin. Knockdown of *PIN1* was confirmed by immunoblotting analysis with anti-Pin1 antibody (Ino et al., 2022b).

## Results

### Characterization of patient-derived PCNSL primary cells

**FIGURE 1:**
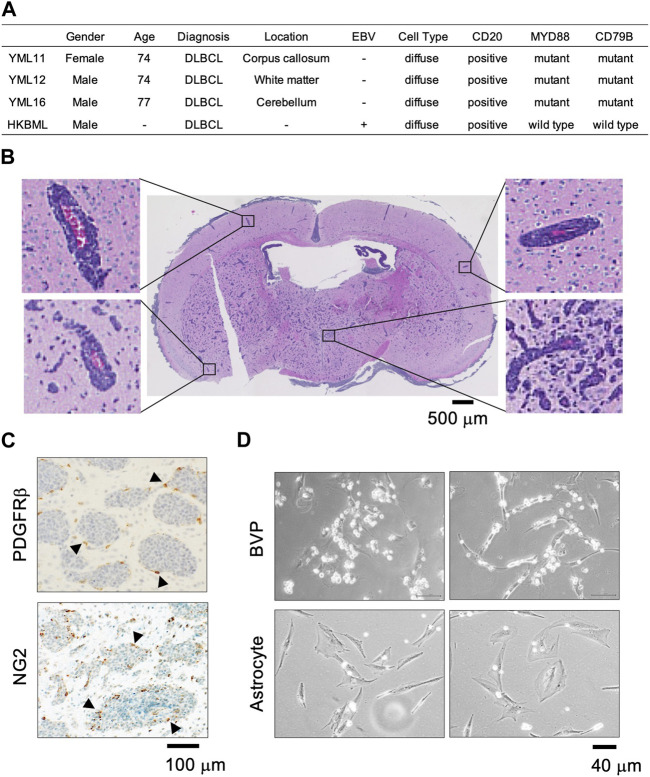
PCNSL cells in the perivascular environment *in vivo* and *in vitro*. **(A)** Clinical, pathological, and genomic information of patients YML11, YML12, and YML16 (PDXs were established at Yokohama City University) and HKBML. **(B)** Hematoxylin and eosin staining of the YML16 orthotopic PDX model. Tumor cells (purple) are located around blood vessels (red). **(C)** Immunohistochemistry analysis of PDGFRβ and NG2 (BVP markers) in the YML16 orthotopic PDX model. Arrowheads indicate cells positive for both PDGFRβ and NG2. BVPs surround tumor cells. **(D)** Phase-contrast images of YML16 cells co-cultured with BVPs or astrocytes. BVPs or astrocytes were cultured in 96-well plates for 3 days, and YML16 cells were seeded on the BVPs for 6 days in PCNSL culture medium. Morphologically, PCNSL cells accumulated and attached directly to BVPs but not to astrocytes.

We previously established PCNSL PDX models (YML11, YML12, YML16) using tumor samples resected by stereotaxic surgery (Tateishi et al., 2020). The EBV status was negative in all three patient samples and corresponding PDX models (Figure 1A). Histopathological analysis of PCNSL xenografts transplanted into brains of immunocompromised mice revealed that tumor cells proliferated predominantly at perivascular sites (Figure 1B), suggesting that BVPs in the tumor microenvironment are important for PCNSL cell survival and proliferation (Grant and Isaacson, 1992). We also performed immunohistochemistry with an anti-PDGFRβ antibody and an anti-NG2 antibody (BVP markers) in PDX model YML16, and found that cells positive for both PDGFRβ and NG2 were surrounded by tumor cells in areas without obvious vascular structures (Figure 1C). We then co-cultured BVPs or primary human brain astrocytes with PCNSL cells in two-dimensional cell cultures and found that the PCNSL cells accumulated and attached directly to the BVPs but not to astrocytes (Figure 1D). These results indicate that the interaction with BVPs may play a role in PCNSL cell survival and proliferation *in vitro*.

### Development of a contacting transwell cell culture system for PCNSL cells and BVPs

**FIGURE 2:**
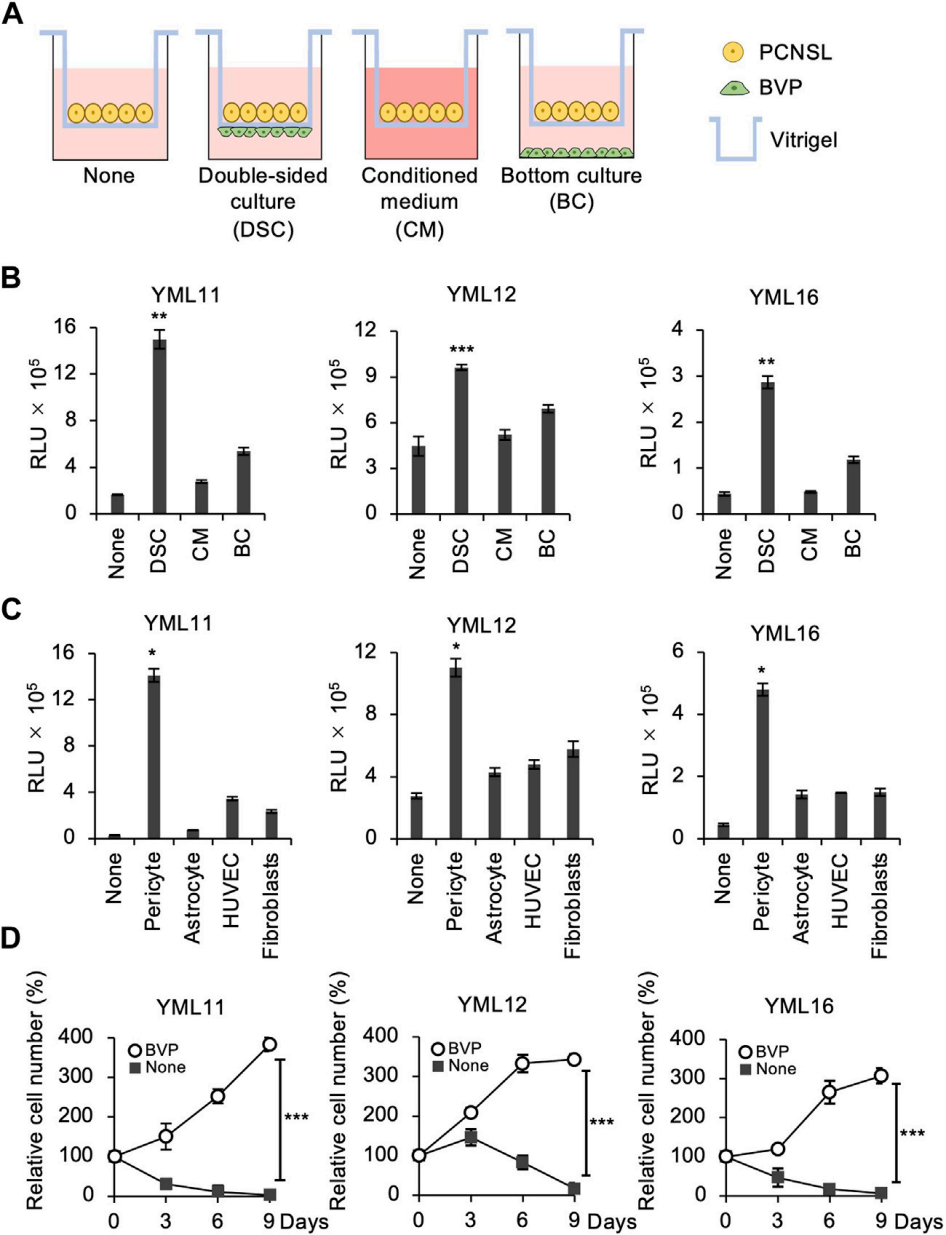
BVPs promote PCNSL cell proliferation in *in vitro* vitrigel culture. **(A)** Experimental design for the vitrigel culture assay. PCNSL cells were cultured on vitrigel only (None), in double-sided culture with BVPs (DSC), with BVP-conditioned medium (CM), or with bottom-cultured BVPs (BC). **(B)** PCNSL cells were cultured under the conditions indicated above for 4 days. The relative cell viability of PCNSL cells was measured after 4 days using CellTiter-Glo. All graphs are presented as mean ± SD (*n* = 3), Welch’s *t*-test (two-tailed), **p < 0.01, ***p < 0.001. **(C)** Vitrigel culture assay of various human epithelial cells. PCNSL cells were co-cultured with pericytes, astrocytes, HUVECs, or fibroblasts. After 4 days, cell viability was measured by CellTiter-Glo. All graphs are presented as mean ± SD (*n* = 3), Welch’s *t*-test (two-tailed), *p < 0.05. **(D)** PCNSL cells were cultured with or without BVPs and harvested at the indicated time points. Relative cell number was measured using CellTiter-Glo. All graphs are presented as mean ± SD (*n* = 3), Welch’s *t*-test (two-tailed), ***p < 0.001.

To reconstruct the tumor microenvironment in the brain, we attempted to establish a layered co-culture of PCNSL cells and BVPs using a collagen vitrigel transwell system (ad-MED-Vitrigel 2), which enables reconstructing crosstalk models between two different cell types (Uzu and Takezawa, 2020). Using this system, PCNSL cells were cultured in three ways: 1) PCNSL and BVP cells were cultured on opposite sides of the insert membrane, i.e., double-sided cell culture (DSC); 2) PCNSL cells were cultured on the top side of the vitrigel in BVP-conditioned medium (CM); and 3) PCNSL cells were co-cultured on the top side of the vitrigel membrane with BVP in the bottom well (BC) below the insert (Figure 2A). Compared to the other two methods, PCNSL cell proliferation was significantly enhanced in the DSC system, thus allowing for extensive cell–cell interactions (Figure 2B). Also, phase-contrast images of PCNSL cells on the vitrigel co-cultured with BVPs showed massive cell proliferation *in vitro* (Supplementary Figure S1A). To identify whether proximity to other cell types had any influence on this proliferation, PCNSL cells were co-cultured in a similar DSC system with other cell species such as astrocytes, human umbilical vein endothelial cells (HUVECs), and embryonic lung fibroblasts. PCNSL cell proliferation was markedly enhanced only in the BVP-DSC system, and not in the systems involving other cell types (Figure 2C). Time-course analysis also revealed favorable growth of PCNSL cells for longer durations in the BVP-DSC system (Figure 2D). By varying the ratio of PCNSL cells to BVPs, we found that a ratio of 1:2 was optimal for PCNSL cell growth (Supplementary Figure S1B).

To examine whether cellular phenotype was sustained during *in vitro* cell culture, we harvested the propagating PCNSL cells from the BVP-DSC system after 15 passages and injected them orthotopically into immunosuppressed mouse brains (Supplementary Figure S2A). We found that these cells retained their original phenotype, characterized by perivascular proliferation (Supplementary Figure S2B) (Tateishi et al., 2020). Furthermore, immunohistochemical analysis demonstrated that the propagated PCNSL cells expressed the B-cell markers CD20 and CD79α, the germinal center markers BCL6, and the activated B-cell marker MUM1, as observed in the original PDXs (Supplementary Figure S2B). These results indicate that PCNSL cells retain their histologic and phenotypic characteristics after long periods of *in vitro* culture and amplification.

### The BVP-DSC system promotes proliferation of EBV-positive PCNSL cells

**FIGURE 3:**
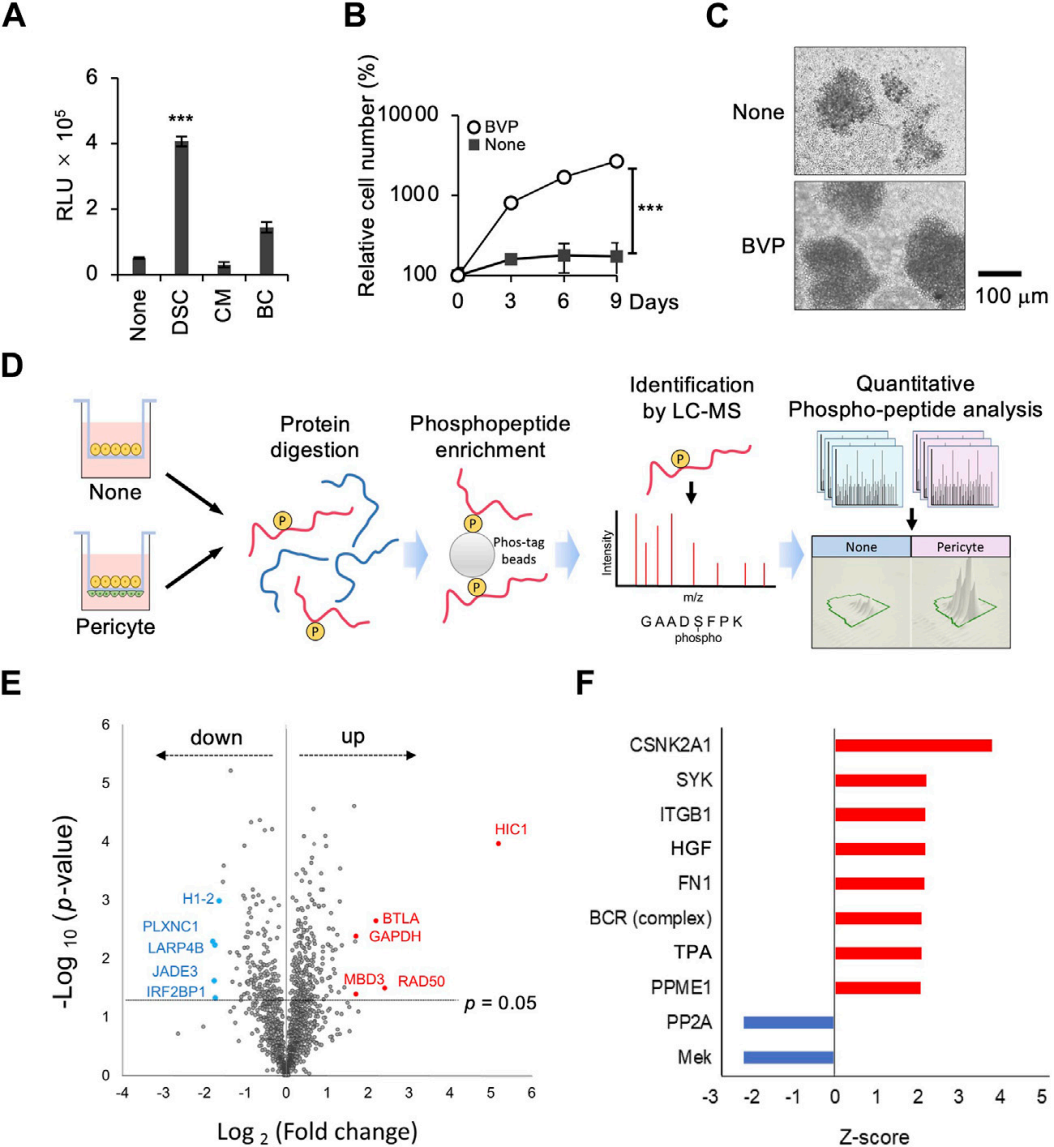
Phosphoproteomic analysis in the interaction between PCNSL cells and BVPs. **(A)** HKBML cells were cultured on vitrigel only (None), in double-sided culture with BVPs (DSC), with BVP-conditioned medium (CM), or with bottom-cultured BVPs (BC). PCNSL cells were cultured under the conditions indicated above for 4 days. The relative cell viability of PCNSL cells was measured after 4 days using CellTiter-Glo. All graphs are presented as mean ± SD (*n* = 3), Welch’s *t*-test (two-tailed), ***p < 0.001. **(B)** HKBML cells were cultured with or without BVPs and harvested at the indicated time points. Relative cell number was measured using CellTiter-Glo. All graphs are presented as mean ± SD (*n* = 3), Welch’s *t*-test (two-tailed), ***p < 0.001. **(C)** Phase-contrast images of HKBML cells on vitrigel co-cultured with or without BVPs. **(D)** Experimental workflow. The effects of co-culture with BVPs on phosphoprotein abundance profiles were evaluated by LC-MS/MS and label-free quantitation. **(E)** Volcano plot representing differences in quantitative phosphoproteomic data. The *x*-axis indicates the log_2_ fold change, and the *y*-axis indicates the −log_10_ *p*-value based on the two-tailed Student’s t-test. The proteins that were significantly upregulated or downregulated under the co-culture condition with BVPs were identified based on two criteria: |fold change| > 2 and *p*-value <0.05. **(F)** List of upstream regulators predicted by phosphoproteomic data–based IPA software analysis to be significantly up- or downregulated in BVP co-culture (*p* < 0.05). The length of the bar indicates the activity prediction *z*-score.

The above results demonstrated that the BVP-DSC system promotes the proliferation of EBV-negative PCSNL cells *in vitro*. We next tested if this system could also enhance proliferation of the EBV-positive PCNSL cell line HKBML. Although HKBML cells can grow independently in monoculture conditions, DSC with BPVs prominently enhanced cell growth compared to the other two methods, as was the case with EBV-negative PCNSL cells (Figure 3A). Time-course analysis showed that cell proliferation in the DSC system was more than 10-fold greater than in the monoculture condition on the vitrigel (Figure 3B). Phase-contrast images showed that co-culture with BVP increased the cell density of HKBML cells compared to monoculture, indicating significant cell proliferation (Figure 3C). The results indicate that a double-sided co-culture system with BVPs also significantly promotes the proliferation of EBV-positive PCNSL cells.

### Molecular interaction between PCNSL cells and BVPs

To further elucidate the molecular mechanism underlying pericyte-dependent PCNSL cell proliferation *in vitro*, we employed a multi-omics approach combining RT-PCR and phosphoproteomic analysis (Supplementary Figure S3). We initially performed a quantitative phosphoproteomic analysis. We collected cell lysates from HKBML cells co-cultured with or without BVPs, and enriched phosphopeptides using Phos-tag agarose beads. We then analyzed these phosphopeptides by LC-MS/MS, and extracted the phosphopeptides that were significantly up- or downregulated (*p* < 0.05) in HKBML cells co-cultured with or without BVPs (Supplementary Table S1). This analysis showed that the expression of 248 proteins was increased while that of 154 proteins was decreased when HKBML cells were co-cultured with BVPs. Volcano plots were generated to identify proteins whose expression differed significantly depending on whether HKBML cells were co-cultured with BVPs. Consequently, we identified 28 upregulated proteins and 20 downregulated proteins as being significantly (fold change > 2, *p* < 0.05) associated with BVP co-culture (Figure 3E).

To further investigate the biological processes affecting PCNSL cell proliferation *in vitro*, we performed an upstream biological analysis within the IPA framework (Kimura et al., 2021). The expression of several phosphorylated proteins was increased or decreased following co-culture with BVPs, and it was postulated that these proteins might be regulated by various intracellular signaling pathways, including those involving hepatocyte growth factor (HGF) and B-cell receptor (BCR) (Figure 3F).

**FIGURE 4:**
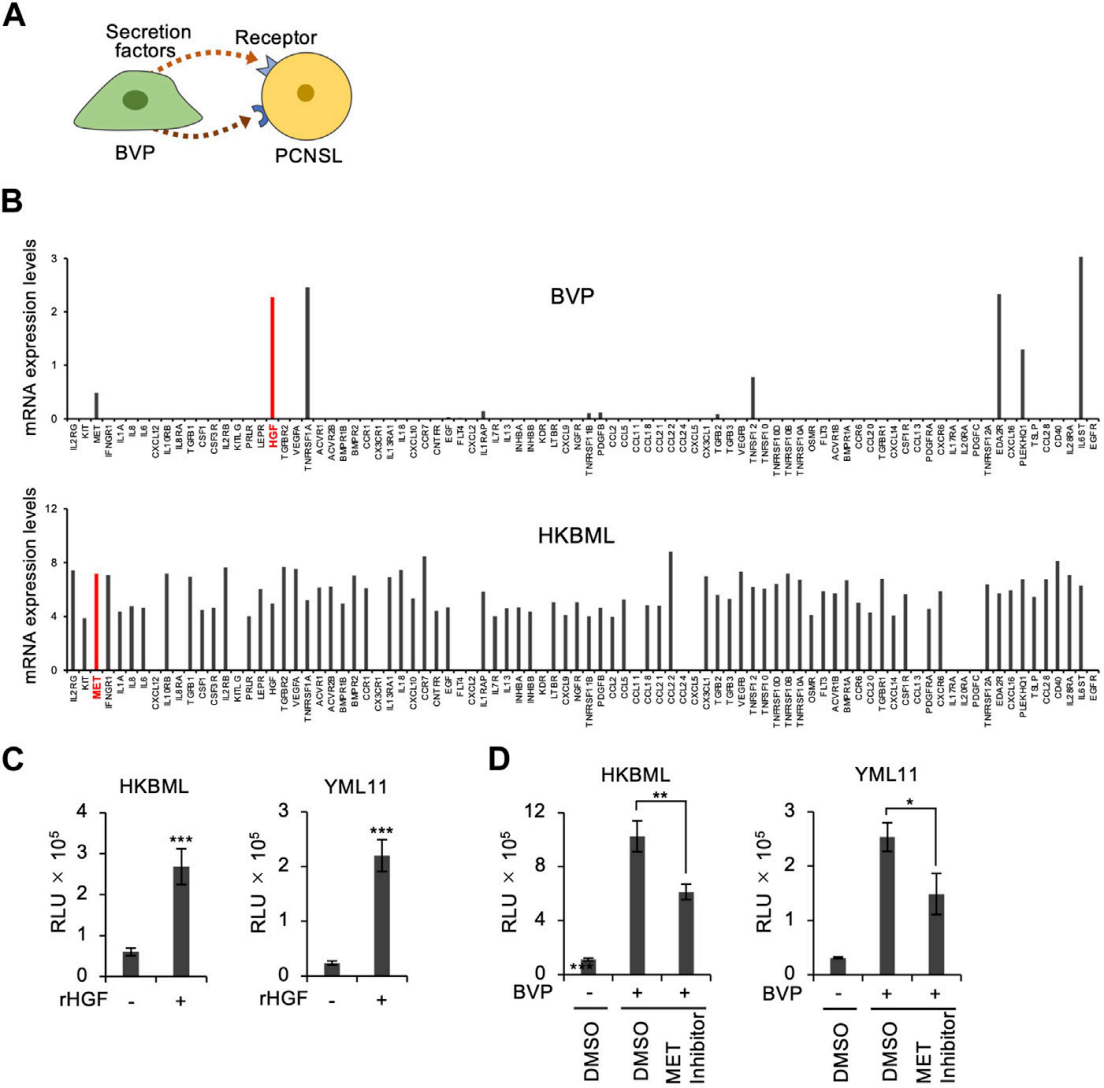
Screening of ligand–receptor interactions between BVPs and PCNSL cells. **(A)** Schematic representation of ligand–receptor interactions between BVPs and PCNSL cells. **(B)** Ligand–receptor interaction screening between BVPs and HKBML cells. Gene expression profiling was performed using a PrimerArray of human cytokine–cytokine receptor interaction (Takara Bio). **(C)** Recombinant human HGF (100 ng/mL) was added after HKBML and YML11 cells were seeded on the vitrigel and cultured for 4 days. Cell viability was measured by CellTiter-Glo. All graphs are presented as mean ± SD (*n* = 3), Welch’s *t*-test (two-tailed), ***p < 0.001. **(D)** PCNSL cells (HKBML and YML11) were treated with the MET inhibitor PHA665752 (6 μM) and cultured with or without BVPs for 4 days. Cell viability was measured by CellTiter-Glo. All graphs are presented as mean ± SD (*n* = 3), Welch’s *t*-test (two-tailed), *p < 0.05, **p < 0.01.

We next adopted an additional approach to investigate the mechanism of BVP-dependent PCNSL cell proliferation. We searched for combinations of BVP-secreted factors involved in intercellular interactions, as well as their corresponding receptors in PCNSL cells. The gene expression of humoral factors secreted by BVPs, and of the receptors for these factors on the plasma membrane of PCNSL cells, were analyzed by real-time RT-PCR with a PrimerArray for cytokine–cytokine receptor interaction. First, we selected five candidate growth factors and cytokines (HGF, TNFRSF1A, EDA2R, PLEKHQ1, IL6ST) whose mRNA expression levels were above the threshold (>1) in BVPs. Next, we examined the expression of their corresponding receptors in PCNSL cells (Figures 4A, B). We found that HGF and its receptor c-Met were expressed in BVP and PCNSL cells, respectively (Figure 4B). Significantly, our phosphoproteomic analysis indicated the relevance of the HGF pathway in the PCNSL-BVP interaction (Figure 3F). We also found that HGF and c-MET mRNA expression was constant during co-culture of BVPs and HKBML cells (Supplementary Figure S5A, S5B). Based on these results, we focused on the HGF–c-MET axis to characterize BVP-dependent PCNSL cell proliferation.

To clarify the role of the HGF–c-Met axis in BVP-mediated cell proliferation, we added HGF directly to the culture medium of PCNSL cells in the absence of BVPs. Interestingly, HGF treatment significantly enhanced the proliferation of PCNSL cells (Figure 4C), although its effect was not as prominent as that observed with the BVP-DSC system. The addition of c-Met inhibitors only partially suppressed PCNSL cell proliferation in the BVP-DSC system (Figure 4D), suggesting that although the HGF–c-Met axis partially contributes to BVP-dependent PCNSL cell proliferation, one or more other molecular pathways may also be involved.

### Involvement of Pin1 in BVP-dependent PCSNL cell proliferation

**FIGURE 5:**
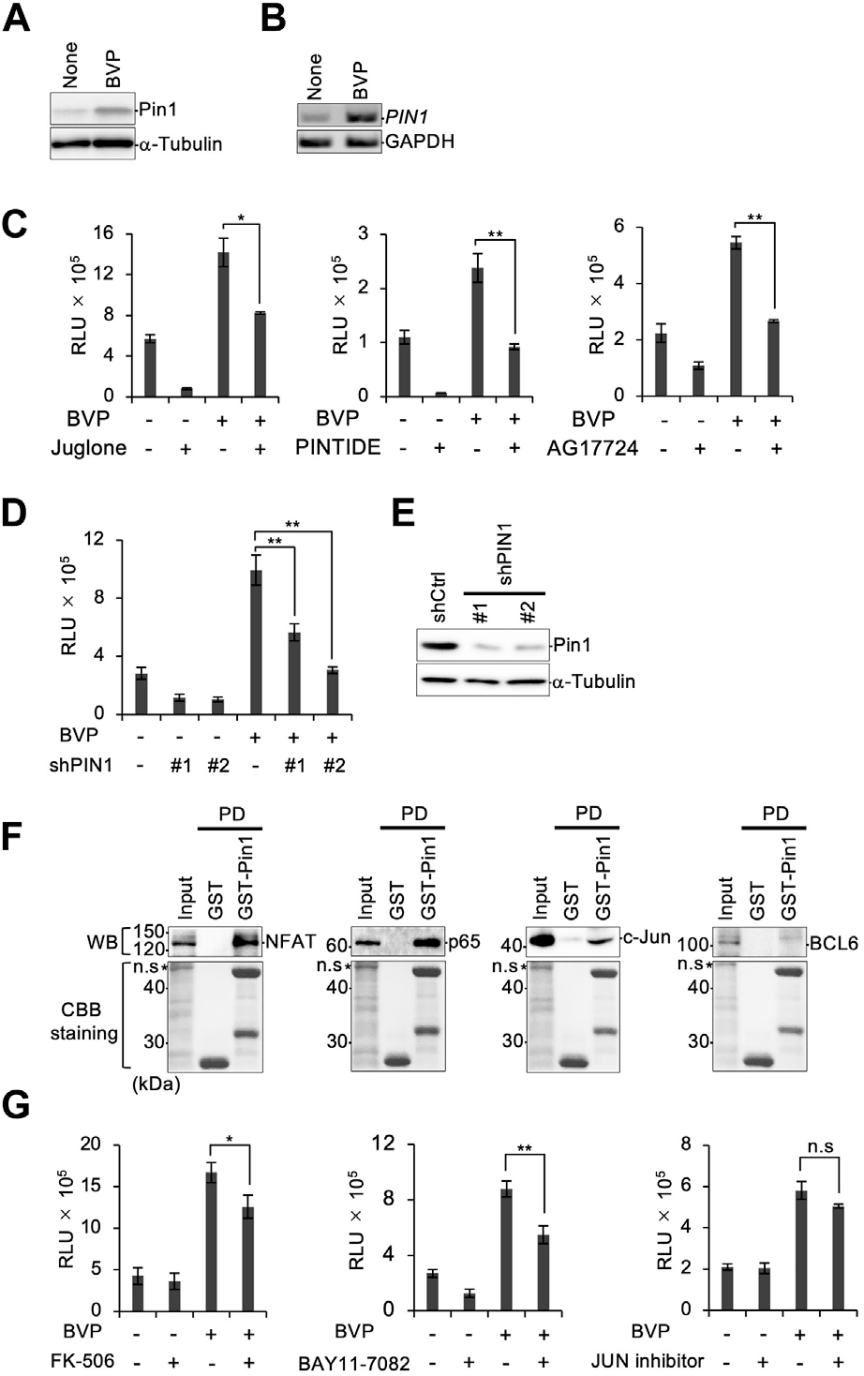
Depletion of Pin1 attenuates PCNSL cell growth. **(A)** HKBML cells co-cultured with or without BVPs for 6 days were collected and the Pin1 expression level was examined by immunoblotting. **(B)** mRNAs were isolated from HKBML cells co-cultured with or without BVPs for 6 days. *PIN1* mRNA was measured by semi-quantitative RT-PCR analysis. GAPDH mRNA is used as a control. **(C)** HKBML cells were seeded on vitrigel with or without BVPs and one of the following Pin1 inhibitors: 50 nM juglone, 40 μg/mL PINTIDE, and 50 μg/mL AG17724. Cells were cultured for 4 days, and cell viability was measured by CellTiter-Glo. All graphs are presented as mean ± SD (*n* = 3), Welch’s *t*-test (two-tailed), *p < 0.05, **p < 0.01. **(D)** HKBML cells were stably transduced with two different shRNAs (#1 and #2) targeting *PIN1*, and shRNA-transduced cells were subjected to a cell viability assay using CellTiter-Glo. All graphs are presented as mean ± SD (*n* = 3), Welch’s *t*-test (two-tailed), **p < 0.01. **(E)** Expression of Pin1 was confirmed by immunoblotting. **(F)** HKBML cells were co-cultured with BVPs, and cell lysates were subjected to GST pull-down analysis with GST or GST-Pin1 followed by immunoblotting with the indicated antibody (top) or Coomassie brilliant blue staining (bottom). **(G)** HKBML cells were seeded with or without BVPs, and with one of the following: 10 µM FK-506 as an NFAT inhibitor, 10 µM BAY11-7082 as an NF-κB inhibitor, and 500 nM JUN inhibitor VIII. Cells were cultured for 4 days, and cell viability was measured by CellTiter-Glo. All graphs are presented as mean ± SD (*n* = 3), Welch’s *t*-test (two-tailed), *p < 0.05, **p < 0.01.

Since we found that the HGF–c-Met axis was only partially responsible for BVP-dependent PCNSL cell proliferation, we sought to identify other factors that might be involved. We decided to investigate the role of the peptidylprolyl isomerase Pin1, since we previously reported that Pin1 enhanced the oncogenic activity and tumor progression of PCNSL (Tateishi et al., 2020). The expression level of Pin1 was increased in PCNSL cells upon co-culture with BVPs (Figure 5A). We also performed semi-quantitative RT-PCR and showed that *PIN1* mRNA was induced by co-culture with BVP (Figure 5B). Furthermore, the Pin1 inhibitors juglone and AG17724, as well as the Pin1-inhibiting peptide PINTIDE, significantly reduced the proliferation of PCNSL cells irrespective of BVP co-culture (Figure 5C), indicating that Pin1 might play an essential role in the proliferation of EBV-positive PCNSL cells *in vitro*. Moreover, targeted shRNA knockdown of *PIN1* significantly reduced the proliferation of PCNSL cells beyond the effect of BVP co-culture (Figures 5D,E). Since HKBML is an EBV-positive PCNSL cell line in which several viral proteins can constitutively activate transcription factors (TFs) such as NF-κB and NFAT (Fu et al., 2006), we tested if Pin1 inhibition affected BVP-dependent cell proliferation in EBV-negative PCNSL cells. Our results showed that Pin1 was induced in EBV-negative YML11 cells upon BVP co-culture, like in HKBML cells, and that targeted Pin1 inhibition indeed suppressed the BVP-dependent cell proliferation in these cells (Supplementary Figure S4A–F).

Pin1 is known to play a central role in phosphorylation signaling by interacting with TFs. We therefore performed GST pull-down assays for NFAT, NF-κB p65, c-Jun, and BCL6, which are reported to be important TFs in B-cell lymphomas. (Cattoretti et al., 2005; Fu et al., 2006; Blonska et al., 2015) (Figure 5F). Our results demonstrated that Pin1 interacted with NFAT, NF-κB p65, and c-Jun, but not with BCL6. We subsequently tested inhibitors targeting the three selective TFs in BVP-dependent PCNSL cell proliferation. Inhibitors of NF-κB and NFAT partially but significantly reduced the proliferation of PCNSL cells following BVP co-culture in both EBV-positive HKBML and EBV-negative YML11 cells (Figure 5G, Supplementary Figure S4F). These results suggest that in addition to BVP signaling involving the HGF–c-Met axis, additional signals mediated by Pin1 via NF-κB and NFAT may influence the survival and proliferation of PCNSL cells.

## Discussion

In this study, we developed a method to culture and propagate patient-derived PCNSL cells *in vitro* using a transwell cell culture system with collagen vitrigel that enables double-sided co-culture with BVPs. This system simulates the tumor microenvironment as it allows the biological activity of BVPs to influence PCNSL cell survival and proliferation *in vitro* without obvious phenotypic changes. Furthermore, we identified the HGF–c-Met axis and Pin1-mediated activation of TFs as the molecular mechanisms underlying the BVP-PCNSL crosstalk that allows PCNSL cells to proliferate while retaining their *in vivo* cellular characteristics.

One of the factors that has hindered effective treatment of PCNSL is the extreme difficulty in establishing cell lines that can be cultured *in vitro*. In particular, EBV-negative PCNSL cells obtained from xenografts rarely proliferate in *in vitro* culture systems. Although commercially available PCNSL cell lines are available (Takashima et al., 2021), there is no detailed information on the donor’s tumor background, and the cell lines may be derived from atypical PCNSL cases. We histopathologically determined the perivascular growth preference of PCNSL cells and developed a DSC transwell co-culture system by reconstructing the brain tumor microenvironment characterized by BVP involvement. We found that our DSC system provided a reliable and reproducible means of propagating PCNSL cells *in vitro* while preserving their original phenotype. This method will allow researchers to elucidate the molecular pathways and mechanisms underlying PCNSL tumorigenesis and will be useful for drug screening against individual patient-derived tumors under physiologically relevant conditions.

Phosphoproteomics and multiplex RT-PCR analysis suggested that HGF secreted from BVPs binds to c-Met on adjacent PCNSL cells, leading to the activation of intracellular signaling. Due to its multifunctional role in oncogenesis and cancer progression, the HGF–c-Met axis has recently been studied extensively in cancer research (Moosavi et al., 2019; Fu et al., 2021). Indeed, c-MET has been shown to be overexpressed in 26.0%–73.2% of cases of diffuse large B-cell lymphoma (Lam et al., 2016), and to be significantly associated with intracellular signaling pathways (Uddin et al., 2010). The c-MET protein structure is characterized by a highly glycosylated 45-kDa extracellular alpha subunit and a 145-kDa transmembrane beta subunit, both of which are linked by disulfide bonds to form mature receptors. Upon binding to HGF, the two MET subunits dimerize and are phosphorylated, leading to autophosphorylation of two other tyrosine residues that are docking sites for downstream signaling molecules related to cell proliferation pathways such as Ras/Raf/MAPK and PI3K/AKT/mTOR (Zhang et al., 2018). HGF is a paracrine cell-growth factor known to induce cell proliferation, cell motility, and morphogenesis (Yi et al., 1998; Nakamura and Mizuno, 2010). It is secreted by mesenchymal cells and acts primarily as a multifunctional cytokine for epithelial cells. c-Met has been shown to be expressed on the plasma membrane of BVPs, and may promote their activity in an autocrine system (Kato et al., 2018). We showed in the present study that HGF secreted from BVPs stimulated c-Met on adjacent PCNSL cells in a paracrine manner. Inhibiting c-Met activity with specific inhibitors suppressed the BVP-enhanced proliferation of PCNSL cells. These results indicate that the HGF–c-Met axis plays a role in the perivascular extension and cell proliferation of PCNSL, hinting at the possibility of exploiting this axis as a target of anti-tumor therapy.

Additionally, our RT-PCR screening indicated the involvement of interleukin-6 (IL-6) in BVP–PCNSL cell crosstalk. Indeed, IL-6 was expressed in PCNSL cells but not in BVPs, and IL-6ST (gp130), a component of the IL-6 receptor (IL-6R), was expressed in both PCNSL cells and BVPs, suggesting that IL-6 may be involved in the intercellular crosstalk. IL-6 is a multifaceted cytokine produced by various cells during inflammation and immunological attack, and it is also relevant to the tumor microenvironment and anti-tumor immunity (Tilg et al., 1994). IL-6 acts through an 80-kDa membrane-bound IL6-R receptor complex composed of two distinct membrane-bound glycoproteins, IL-6R and gp130. After IL-6 binds to IL-6R, the IL-6/IL-6R complex associates with gp130 and activates JAK–STAT signaling (Heinrich et al., 1998). Previous reports have shown that IL-6 not only mediates inflammation but also exerts a neuroprotective effect by stabilizing pericytes in the setting of cerebral ischemia (Nakamura et al., 2012). Furthermore, IL-6 can provide critical intracellular signaling necessary for growth and survival of diffuse large B-cell lymphoma (Hashwah et al., 2019). Further analysis is needed to delineate the biological function of the IL-6/IL-6R axis in BVP–PCNSL cell crosstalk.

The peptidylprolyl isomerase Pin1 regulates a subset of phosphoproteins by catalyzing the *cis-trans* isomerization of the phosphorylated Ser/Thr-Pro motif (Lu et al., 1996; Lu et al., 2002; Yaffe et al., 1997). Pin1 is overexpressed in many types of human malignancies and has been implicated in several oncogenic signaling pathways that enhance or occasionally suppress tumor progression (Ryo et al., 2001; 2003; 2005; 2007). We showed in our previous study that Pin1 plays a crucial role in PCNSL progression by mediating the constitutive activation of RelA/p65 (Tateishi et al., 2020). Pin1 has been shown to interact with several substrate proteins in a phosphorylation-dependent manner (Chen et al., 2018). Therefore, Pin1 can be used as a molecular probe to capture cancer-specific phosphoproteins responsible for the oncogenesis and sustained malignant properties of cancers. In the current study, we further delineated the roles of Pin1 in the survival and proliferation of PCNSL cells *in vitro*. This study demonstrated that Pin1 is upregulated in PCNSL cells upon co-culture with BVPs. Although it remains unclear how BVPs induce Pin1 upregulation, previous reports have shown that Pin1 expression is enhanced by several transcription factors, including E2F, Notch, and C/EBP (Ryo et al., 2002; Rustighi et al., 2009; Pulikkan et al., 2010), as well as cytokines such as IL-22 and IL-33 (Chen et al., 2018; Nechama et al., 2018). More detailed analysis is needed to elucidate the full molecular basis of Pin1 gene expression during the BVP–PCNSL cell crosstalk.

Our phosphoproteomic analysis showed that PCNSL cells exhibited upregulation of phosphoproteins involved in BCR signaling when co-cultured with BVPs. Indeed, antigen-mediated triggering of the BCR has been shown to activate both NF-κB and NFAT (Fu et al., 2006). Antigen binding activates the BCR and phosphorylates immunoreceptor tyrosine-based activation motifs (ITAMs) in the cytoplasmic domains of CD79A and CD79B, thereby leading to the autophosphorylation of Syk and further phosphorylation of ITAMs (Rolli et al., 2002). The steady-state activation of the BCR induces a series of phosphorylation signaling pathways, thereby leading to both NFAT and NF-κB activation, which has been implicated in long-term biological responses such as the proliferation, survival, and differentiation of B lymphocytes (Fu et al., 2006). A subset of PCNSLs have acquired genetic mutations in CD79B or MYD88, thereby constitutively activating the NF-κB pathway (Tateishi et al., 2020). Activation of NF-κB signaling is a hallmark of PCNSLs with recurrent mutations in CD79B or MYD88. These tumors generally exhibit chronically activated BCR signaling and induce constitutive activation of the NF-κB pathway. Interestingly, previous studies involving large cohorts of PCNSL patients receiving a uniform chemotherapy regimen of rituximab, methotrexate, procarbazine, and vincristine (R-MPV) indicated that the MYD88 L265P and CD79B Y196 mutations were associated with prognosis (Nakamura et al., 2016; Nayyar et al., 2019). The exact molecular link between BCR signaling and the HGF–c-Met axis is still unclear. However, it was shown that c-Met inhibition reduces constitutive NF-κB activity in solid tumors such as esophageal adenocarcinoma and liver cancer (Watson et al., 2006; Li et al., 2019). Further analysis is needed to identify potential therapeutic strategies targeting the functional crosstalk of these molecules.

Although the main limitation of this study is the small number of cell lines used in the experiments, it should be noted that co-culture with pericytes promoted the growth of not only EBV-negative cells (YML11, YML12, YML16), but also EBV-positive cells (HKBML). In addition, we focused on only a few factors involved in the enhancement of PCNSL cell growth and proliferation by co-culture. EBV-positive B-cell lymphomas exhibit constitutive activation of TFs, including NF-κB and NFAT (Fu et al., 2006), leading to the transformation of B cells (Price and Luftig, 2014). Our current study showed that EBV-positive HKBML cells proliferated in the absence of BVP co-culture, albeit partially and slowly, representing BVP-independent cell proliferation. Since Pin1 is essential for the activation of these TFs, targeted inhibition of Pin1 resulted in cell growth suppression even in the absence of BVPs. However, BVP co-culture may promote cell proliferation by fully activating the intracellular signaling, which is also suppressed by Pin1 inhibition. On the contrary, BVP co-culture is essential for the proliferation of EBV-negative PCNSL cells *in vitro*, probably due to the absence of basal activation of TFs. Indeed, BVP co-culture resulted in the TF activation, which was canceled by Pin1 inhibition. Therefore, Pin1 may only be involved in BVP-dependent cell proliferation in EBV-negative PCNSL cells. Over 100 factors have been reported to bind to Pin1, and one or more of these may play a role as well. We demonstrated that inhibition of PCNSL cell growth by Pin1 inhibition was more pronounced than that resulting from inhibition of other factors, making it likely that Pin1 activates additional factors; however, further analysis is needed to elucidate the precise molecular mechanisms of this co-culture model. Despite these limitations, the co-culture model developed in this study shows promise as a versatile system for culturing primary PCNSL cells that have been surgically isolated from patients, and for testing the therapeutic efficacy of drugs to formulate a personalized treatment protocol using agents to which the cells respond *in vitro*. Unresolved questions pertaining to PCNSL pathophysiology and therapeutic development may be addressed using this cell culture model alongside drug discovery and multi-omics technologies.

## Data Availability

The datasets generated and/or analyzed during the current study are available using the following link - https://proteomecentral.proteomexchange.org/cgi/GetDataset?ID=PXD043128.

## Abbreviations

PCNSL, primary CNS lymphoma; BVPs, brain vascular pericytes; DLBCL, diffuse large B-cell lymphoma; EBV, Epstein Barr virus; Pin1, peptidyl-prolyl cis-trans isomerase NIMA-interacting 1; PDX, patient-derived xenograft; PDGFRβ, platelet-derived growth factor receptor; HUVEC, human umbilical vein endothelial cells; HGF, hepatocyte growth factor; BCR, B-cell receptor; DSC, double-sided cell culture; CM, culture in conditioned medium; BC, culture in the bottom well with a top well; GST, glutathione S-transferase; LC-MS/MS, liquid chromatography/tandem mass Spectrometry; RT-PCR, reverse transcriptase-polymerase chain reaction; ITAM, immunoreceptor tyrosine-based activation motif; Syk, spleen tyrosine kinase; NFAT, nuclear factor of activated T cells; NF-κB, nuclear factor-κB.
