# Supplementary figures and images for: Development of a contacting transwell co-culture system for the in vitro propagation of primary central nervous system lymphoma
Source: Front Cell Dev Biol. 2023 Nov 27;11:1275519. doi: 10.3389/fcell.2023.1275519 (PMC10712316; doi:10.3389/fcell.2023.1275519)

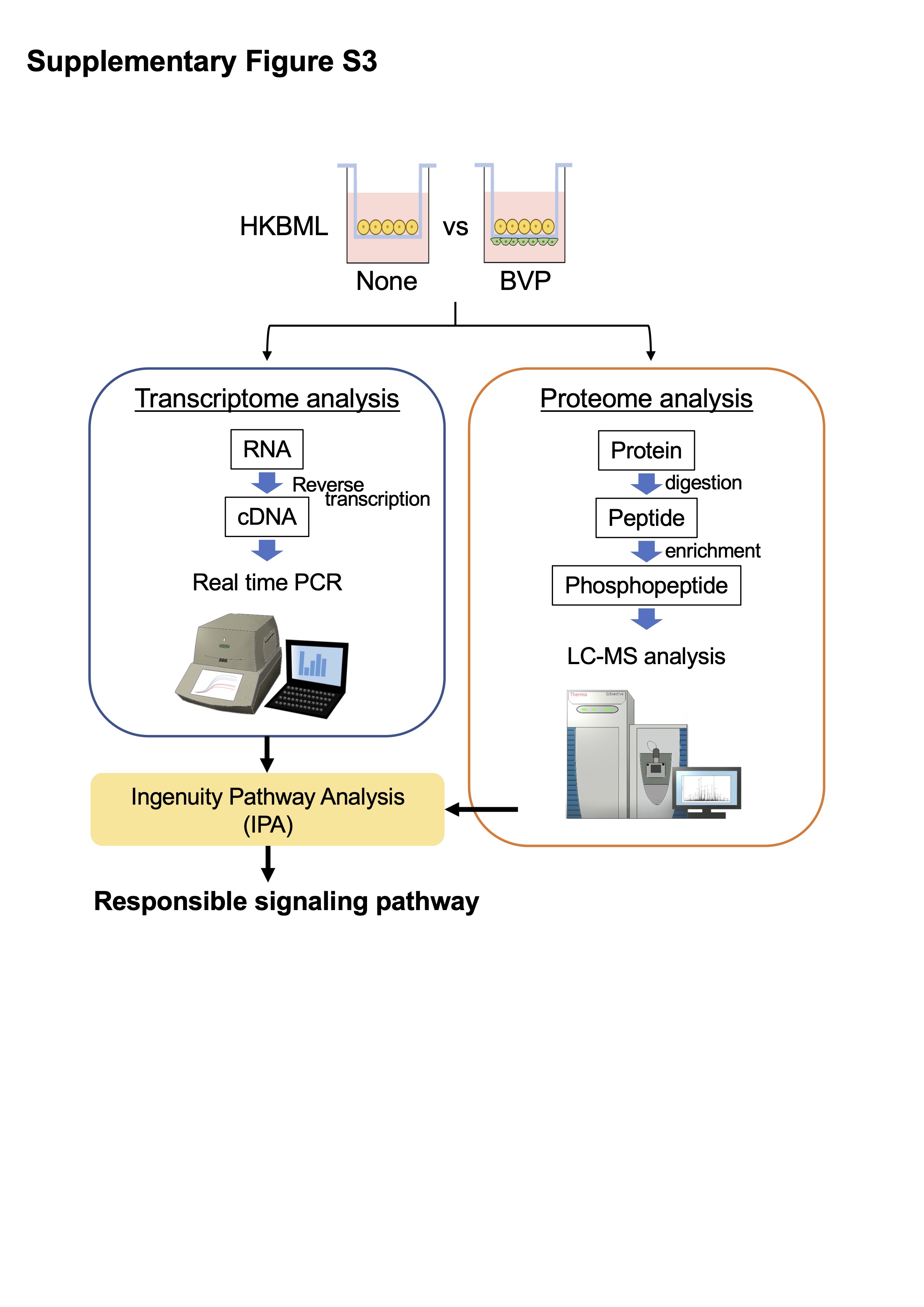

Supplement: Supplementary file 1 [file Image3.jpeg]

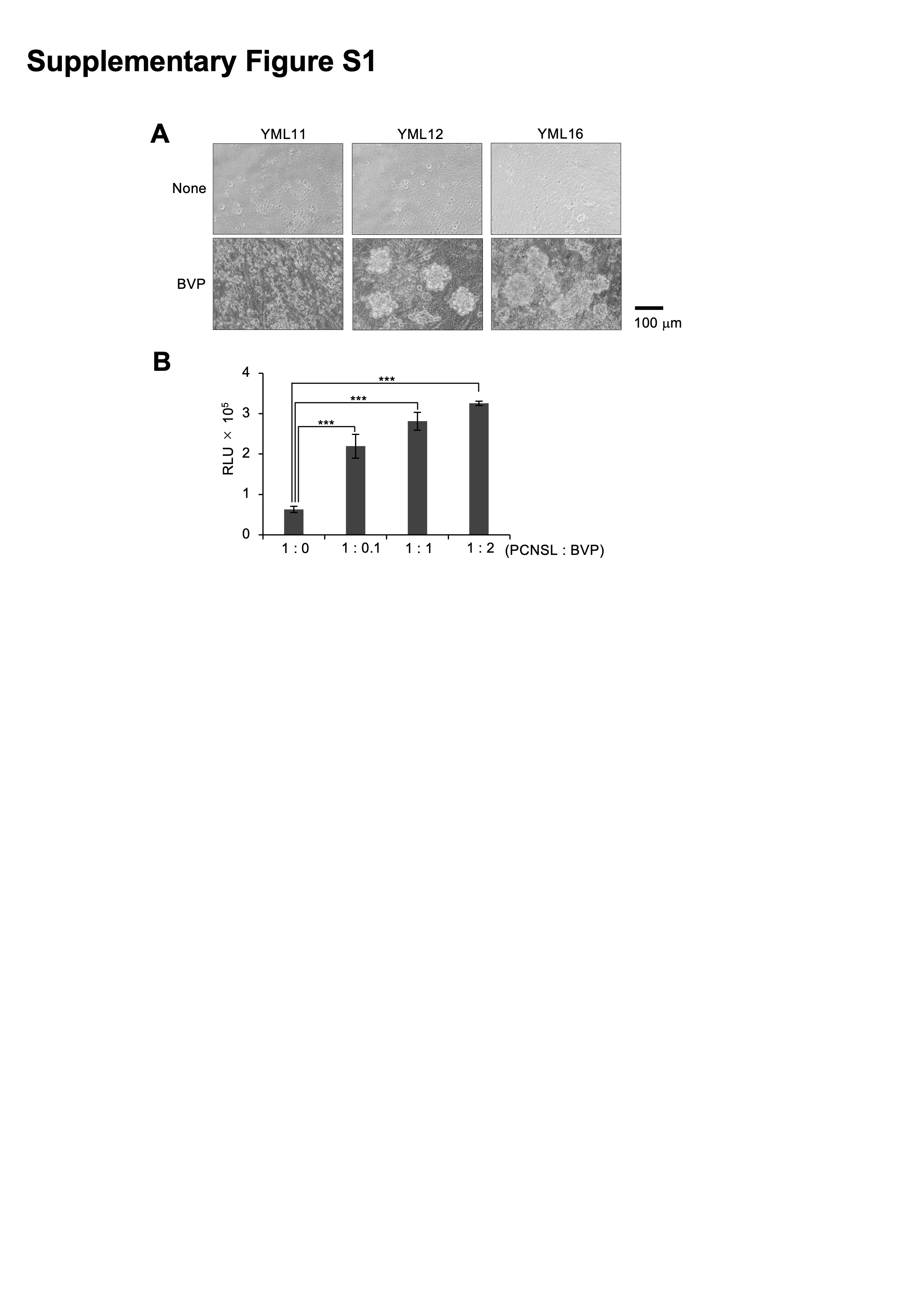

Supplement: Supplementary file 3 [file Image1.jpeg]

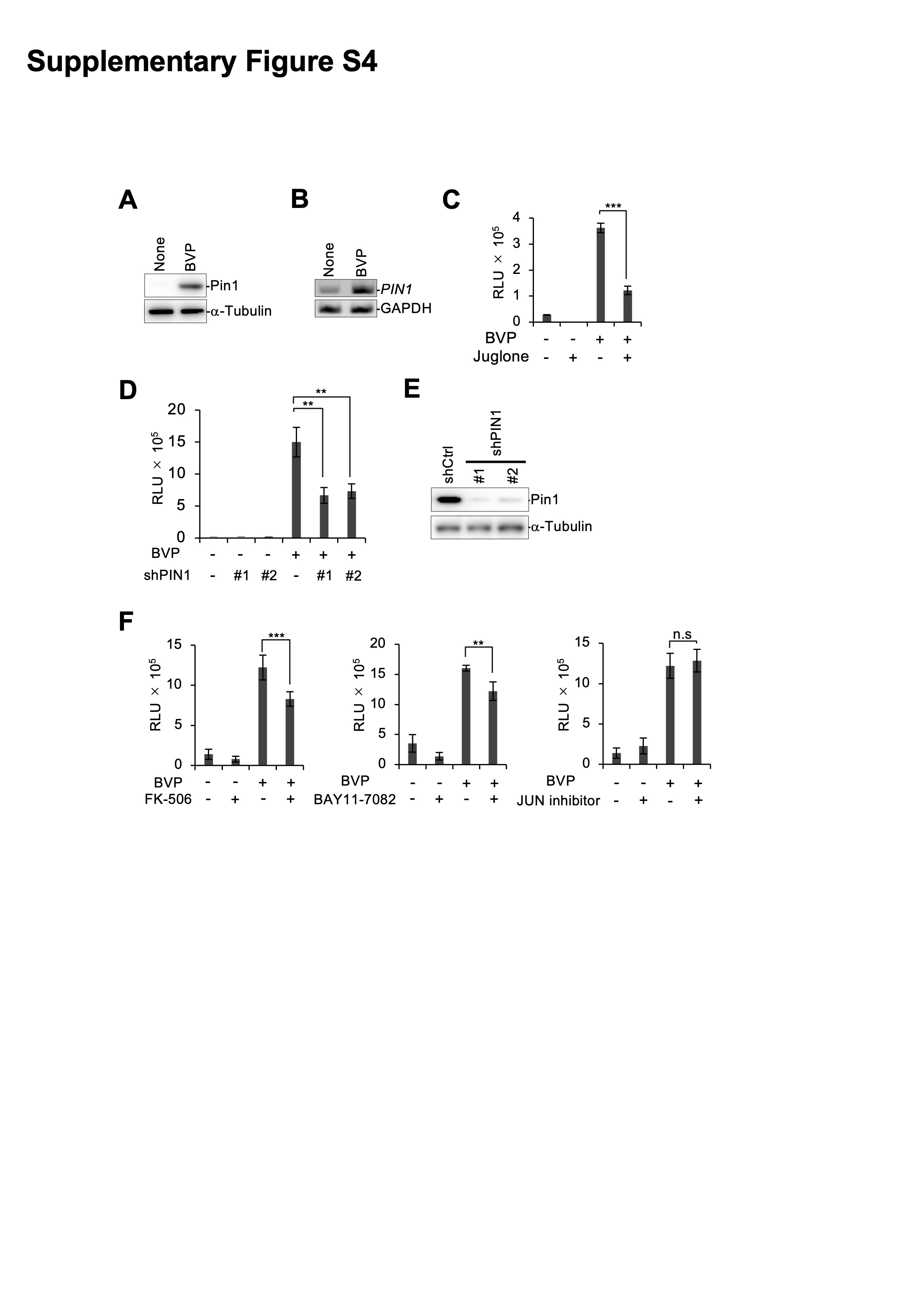

Supplement: Supplementary file 4 [file Image4.jpeg]

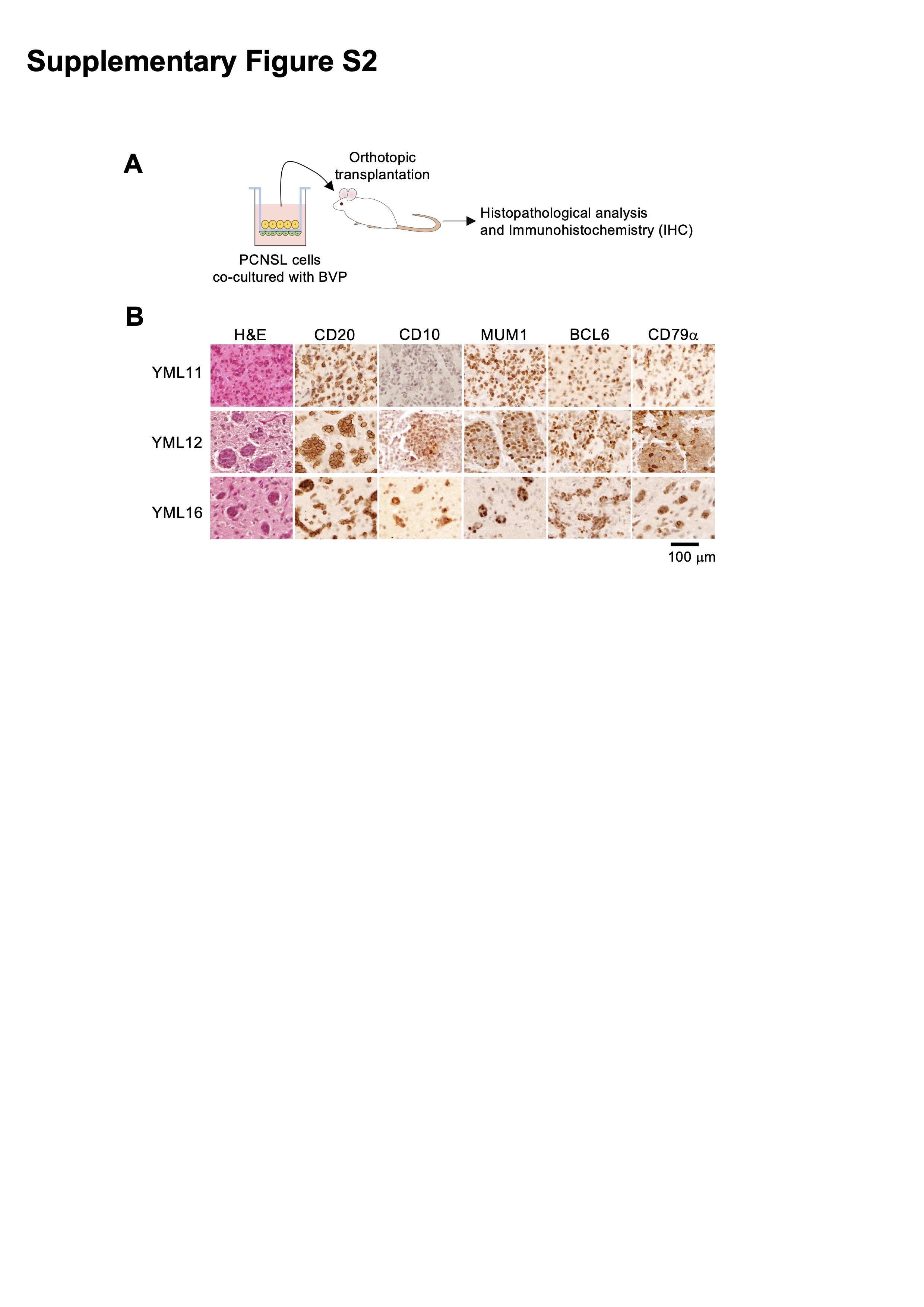

Supplement: Supplementary file 5 [file Image2.jpeg]

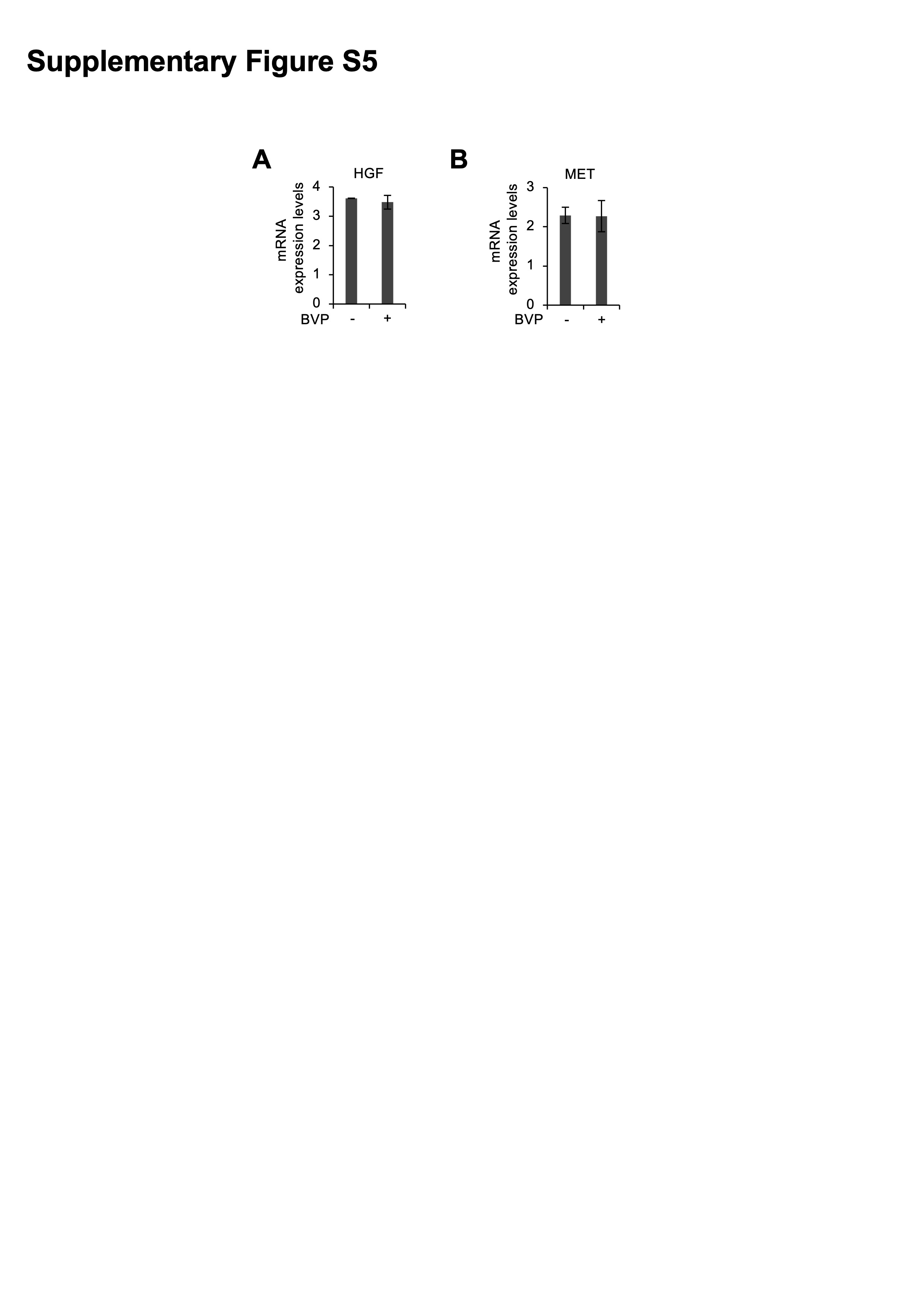

Supplement: Supplementary file 6 [file Image5.jpeg]
