# Supplementary material for: Development of a contacting transwell co-culture system for the in vitro propagation of primary central nervous system lymphoma
Source: Front Cell Dev Biol. 2023 Nov 27;11:1275519. doi: 10.3389/fcell.2023.1275519 (PMC10712316; doi:10.3389/fcell.2023.1275519)
Supplement: Supplementary file 2 [file Table1.pdf]

Supplementary Table S1

Upstream Regulator	Molecule Type	Predicted Activation State	Activation z-score	*p*-value of overlap	Target Molecules in Dataset
CSNK2A1	kinase	Activated	3.795	2.38E-13	ATP2B1,CANX,CCDC6,CD2BP2,DDX54,EEF1D,EIF3J,EIF5B,FNBP4,FTSJ3,HDAC1, HDAC2,HIRIP3,HNRNPC,HTATSF1,KRI1,MCM3,MFAP1,MPHOSPH10,NCAPD2,NPM1, PDIA6,PML,PRCC,RSF1,RSL1D1,SEPTIN2,SF3A1,SRSF10,SRSF2,SSB,SUPT5H, SURF6,TMPO,USP7,WDR44,XRCC4,XRN2,ZC3H15,ZRANB2
SYK	kinase	Activated	2.204	0.184	CFL1,CTTN,EP300,ITSN2,LCP2,MAP4K1,PTK2B,TUBA1C
ITGB1	transmembrane receptor	Activated	2.2	0.0994	BCAR1,CFL1,CTTN,LCP2,PTK2B
HGF	growth factor	Activated	2.191	0.456	BCAR1,CTTN,EPS15,HNRNPC,MVP,NCOA3,NPM1,PAK1,RB1,TNIK
FN1	enzyme	Activated	2.16	1	BCAR1,CFL1,DNMT1,HNRNPC,LSP1,MATR3,PGAM1,PRPF4B,PTK2B,RPS2,TJP2
BCR (complex)	complex	Activated	2.106	0.1	BCAR1,CARD11,CD22,ITSN2,LCP2,PRKCB,PTK2B,RB1,RPS6,TUBA1C
tetradecanoylphorbol acetate (TPA)	chemical drug	Activated	2.09	0.0194	ARHGEF2,ARID1A,BCAR1,CARD11,CD44,CD74,CFL1,CTTN,FERMT3,H1-2,LCP1,MARCKS, NCOR1,OPTN,PITPNM1,PRKCB,PTK2B,RB1,RPS6,SP3,SPHK2,TTF1,UFD1,WDR44
PPME1	enzyme	Activated	2.067	0.000000242	ATRX,DDX41,HDAC2,HDAC4,KDM1A,LMNA,NPM1,PAXBP1,SRSF9,TMPO,TP53BP1
PP2A	complex	Inhibited	-2.191	1	ACACA,ATXN1,CAMK4,CARD11,UPF1
Mek	group	Inhibited	-2.201	0.13	CCAR2,ELAVL1,PRKDC,RB1,RPS6
